# Impact of Percent Body Fat on All-Cause Mortality among Adequate Dialysis Patients with and without Insulin Resistance: A Multi-Center Prospective Cohort Study

**DOI:** 10.3390/nu11061304

**Published:** 2019-06-09

**Authors:** Tuyen Van Duong, Te-Chih Wong, Hsi-Hsien Chen, Tso-Hsiao Chen, Yung-Ho Hsu, Sheng-Jeng Peng, Ko-Lin Kuo, Hsiang-Chung Liu, En-Tzu Lin, Shwu-Huey Yang

**Affiliations:** 1 School of Nutrition and Health Sciences, College of Nutrition, Taipei Medical University, Taipei 110, Taiwan; duongtuyenvna@gmail.com; 2 Department of Nutrition and Health Sciences, Chinese Culture University, Taipei 110, Taiwan; wdz5@ulive.pccu.edu.tw; 3 Division of Nephrology, Department of Internal Medicine, School of Medicine, College of Medicine, Taipei Medical University, Taipei 110, Taiwan; 570713@yahoo.com.tw (H.-H.C.); tsohsiao@tmu.edu.tw (T.-H.C.); yhhsu@tmu.edu.tw (Y.-H.H.); 4 Division of Nephrology, Department of Internal Medicine, Taipei Medical University Hospital, Taipei 110, Taiwan; 5 Department of Nephrology, Taipei Medical University-Wan Fang Hospital, Taipei 110, Taiwan; 6 Division of Nephrology, Department of Internal Medicine, Taipei Medical University—Shuang Ho Hospital, Taipei 110, Taiwan; 7 Division of Nephrology, Cathay General Hospital, Taipei 110, Taiwan; kennethpeng@cgh.org.tw; 8 Division of Nephrology, Taipei Tzu-Chi Hospital, New Taipei 231, Taiwan; kolinkuo8@gmail.com; 9 Department of Nephrology, Wei Gong Memorial Hospital, Miaoli 351, Taiwan; hc88liu@gmail.com; 10 Department of Nephrology, Lotung Poh-Ai Hospital, Yilan 265, Taiwan; nick.et.lin@gmail.com; 11 Research Center of Geriatric Nutrition, Taipei Medical University, Taipei 110, Taiwan; 12 Nutrition Research Center, Taipei Medical University Hospital, Taipei 110, Taiwan

**Keywords:** percent body fat, obesity, insulin resistance, HOMA-IR, all-cause mortality, hemodialysis, mortality, survival, dialysis adequacy, multi-center

## Abstract

The association between body fat and mortality in hemodialysis patients remains controversial. We examined the effect of percent body fat (PBF) on all-cause mortality among adequate hemodialysis patients with and without insulin resistance (IR). A prospective cohort study was conducted on 365 adequate hemodialysis patients (equilibrated Kt/V ≥ 1.2) from seven hospitals. Patients’ characteristics and clinical and biochemical parameters were assessed at baseline between September 2013 and April 2017. Patients were followed up for all-cause mortality until April 2018. The median value of homeostatic model assessment (HOMA-IR) was used to classify IR. Cox proportional hazard models were utilized to examine predictors of all-cause mortality. During 1.4 (1.0–3.2) years of follow-up, 46 patients died. In patients with IR (HOMA-IR ≥ 5.18), PBF was significantly higher in the survival group than in the death group (31.3 ± 9.0 vs. 25.4 ± 8.2, *p* = 0.005). After controlling for confounding factors, PBF was significantly associated with lower risk for all-cause mortality in patients with IR (hazard ratio, 0.94; 95% confidence interval, 0.89–1.00; *p* = 0.033). The association was not observed in patients without IR. In conclusion, percent body fat shows a protective effect on survival in hemodialysis patients with IR.

## 1. Introduction

Obesity is increasing across the globe. It is a strong predictor of metabolic syndrome [1], cardiovascular diseases (CVD), and mortality in different populations [2,3,4]. Conversely, in end-stage renal disease (ESRD) patients undergoing hemodialysis, the “obesity paradox” is well-reported [5,6,7]. It appears that higher body fat, such as higher body mass index [7], and higher level of abdominal fat (e.g., visceral fat, and subcutaneous fat area) are associated with lower mortality in hemodialysis patients [8].

Insulin resistance (IR) is highly prevalent and well-known as a predictor of cardiovascular disease [9], and mortality in ESRD patients [10]. In addition, poor glycemic control is significantly associated with high risk of cardiovascular and all-cause mortality in maintenance hemodialysis patients [11]. It becomes more complicated if hemodialysis patients have abnormalities in glucose metabolism [12]. Therefore, novel approaches in insulin resistance treatment and management are set as important targets for improving clinical outcomes and preventing excess death in CKD patients [13], and ESRD patients treated with hemodialysis [12,14].

Hemodialysis patients with obesity have a survival advantage, which has been reported in empirical studies [15,16,17] and in the meta-analysis [18]. However, most of the previous studies have looked at body mass index as a measure of body size, which does not distinguish between lean body mass and fat mass. A direct measure of body fat mass has been used in a limited number of studies. In addition, the protective effect of body fat among hemodialysis patients with insulin resistance remains to be explored. Therefore, we aimed to examine the effect of percent body fat on all-cause mortality in hemodialysis patients with and without insulin resistance. We hypothesized that higher percent body fat is associated with lower risk of death in patients with insulin resistance.

## 2. Materials and Methods

### 2.1. Study Design and Settings

We conducted a prospective cohort study in seven dialysis centers of Taipei Medical University Hospital, Wan Fang Hospital, Shuang Ho Hospital, Cathay General Hospital, Taipei Tzu-Chi Hospital, Wei-Gong Memorial Hospital, and Lotung Poh-Ai Hospital.

### 2.2. Patient Recruitments

**Figure 1:**
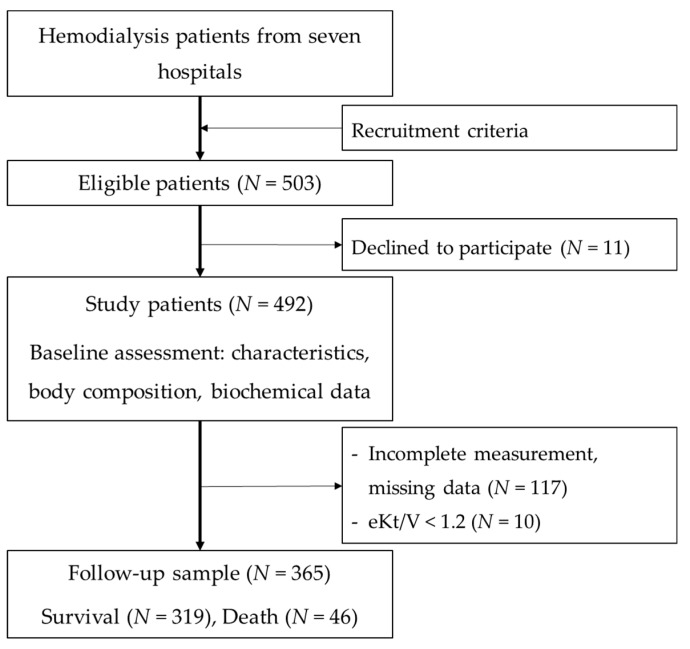
Flow chart of patient sampling and study procedure.

Patients aged 20 years and above, receiving three hemodialysis sessions a week for at least three months were included. Patients were excluded from the study if they were diagnosed with edema, pregnancy, amputation, hyperthyroidism, hypothyroidism, malignancy, received tube feeding, exhibited hepatic failure or cancer, had been hospitalized within one month prior to the recruitment, or had scheduled surgery. Patients with inadequate dialysis quality (equilibrated Kt/V < 1.2 g/kg/day) at the recruitment or during the following-up were also excluded. Patients’ recruitment criteria was also described previously [19]. At baseline (September 2013–April 2017), patients’ characteristics and clinical and biochemical parameters were examined. Patients were then followed up for all-cause mortality until April 2018. A final sample of 365 patients were followed up and analyzed, which is illustrated in Figure 1.

### 2.3. Patients’ Characteristics

Patients’ age, gender, hemodialysis vintage, and Charlson comorbidity index [20] were assessed using chart review. The short version of the International Physical Activity Questionnaire was used to assess physical activity level [21]. Patients were asked about their time spent on vigorous, moderate, walking, and sitting activities over the last seven days. Physical activity was estimated using the metabolic equivalent task scored in minutes per week (MET-min/week) [22]. The number of MET-min/week was the sum of minutes that patient spent at each level, multiplied by 8.0, 4.0, 3.3, and 1.0, respectively [23].

### 2.4. Clinical Parameters

Systolic blood pressure (SBP), diastolic blood pressure (DBP), body mass index (BMI), and cardiothoracic ratio (CTR) were collected using medical records. Body composition, including total muscle mass (TMM), body fat mass (BFM), and percent body fat (PBF), was assessed using a bioelectrical impedance analysis (BIA) device using multiple operating frequencies of 1, 5, 50, 250, 500, and 1000 kHz (InBody S10, Biospace, Seoul, Korea).

### 2.5. Biochemical Parameters

Blood samples at the first dialysis session were collected by registered nurses and analyzed in the hospital laboratory using the standard protocol. The biochemical parameters were high sensitive C-reactive protein (hs-CRP), hemoglobin (Hgb), fasting plasma glucose (FPG), fasting plasma insulin (FPI), triglyceride (TG), high density lipoprotein cholesterol (HDL-C), low density lipoprotein cholesterol (LDL-C), total cholesterol (TC), serum calcium (Ca), serum phosphate (PO_4_), intact parathyroid hormone (iPTH), homocysteine (Hcy), albumin, pre-dialysis blood urea nitrogen (Pre-BUN), creatinine, serum potassium (K), and serum uric acid (SUA).

The homeostatic model assessment index (HOMA-IR) was used to assess insulin resistance. The index was calculated using the formula developed by Matthews et al. [24]:

HOMA-IR = fasting plasma insulin (μU/mL) × fasting plasma glucose (mg/dL)/405.

The median value of HOMA-IR was used to classify patients into groups with and without insulin resistance. This classification was also applied in previous studies [10,25].

Biochemical parameters were classified as inflammation (hs-CRP > 0.5 mg/dL) [26], anemia (Hgb < 11 g/dL) [27], impaired fasting glucose (IFG) (FPG ≥ 100 mg/dL, diagnosed type 2 diabetes (T2DM)), or high insulin (FPI ≥ 12 µU/mL) [28]. Dyslipidemia was classified as TG ≥ 150 mg/dL, or HDL-C < 40 mg/dL for men, or HDL-C < 50 mg/dL for women, or LDL-C ≥ 100 mg/dL, or TC ≥ 200 mg/dL [29,30,31,32]. Serum calcium was classified into normal (Ca ≤ 9.5 mg/dL) vs. high (Ca > 9.5 mg/dL). Levels of PO_4_ were classified into normal (PO_4_ ≤ 5.5 mg/dL), vs. high (PO_4_ > 5.5 mg/dL). Calcium–phosphorus combination was classified into normal (Ca x PO_4_ < 55 mg^2^/dL^2^) vs. high (Ca x PO_4_ ≥ 55 mg^2^/dL^2^). Levels of iPTH were classified into normal (iPTH 150–300 pg/mL) vs. high (iPTH ≥ 300 pg/mL) [33]. Homocysteine > 14 µmol/L was defined as hyperhomocysteinemia [34]. Serum potassium ≥ 5.0 mEq/L was defined as hyperkalemia [35]. Albumin, pre-dialysis blood urea nitrogen (Pre-BUN), creatinine, and serum uric acid (SUA) were analyzed as continuous variables.

### 2.6. Ethical Consideration

The study protocol was approved by the Ethics Committees (JIRB No. 201302024; OP104001; 04-M11-090). Patients signed informed consent forms before the study conducted.

### 2.7. Statistical Analysis

The distribution of continuous variables was tested using a Shapiro–Wilk test (normal if *p*-value > 0.05) [36,37], and a visual inspection of their histograms, normal Q-Q plots and box plots was made. The variables with approximately normal distributions were reported as mean ± SD, otherwise median (interquartile range) is reported. The categorical variables were presented as frequency and percentages. The *t*-test, Mann-Whitney *U* test, or the Chi-Square test were used to compare the distribution of characteristics and clinical and biochemical parameters between the survival and death groups, respectively.

The associations of patients’ characteristics and clinical and biochemical parameters with all-cause mortality in the overall sample and subgroups of HOMA-IR were analyzed using bivariate Cox proportional hazard models. Furthermore, the protective effect of percent body fat on survival in patients with and without insulin resistance was examined using multivariate Cox proportional hazard models. The adjusted variables were those with significant effects on all-cause mortality in the bivariate model, appropriately. Hazard ratios and 95% confidence intervals were reported.

Data were analyzed using the IBM SPSS software version 20.0 for Windows (IBM Corp., New York, NY, USA). The significant level was set at *p*-value < 0.05.

## 3. Results

During a median of 1.4 years (ranged 1.0–3.2) of follow-up, 46 (12.6%) deaths were reported. The median value of HOMA-IR was 5.18. The patients who died were those with higher Charlson comorbidity index (*p* < 0.001), lower physical activity level (*p* = 0.002), higher CTR (*p* = 0.004), higher hs-CRP (*p* < 0.001), higher prevalence of IFG (*p* = 0.037), lower serum creatinine (*p* = 0.017), and lower serum uric acid (*p* = 0.021; Table 1).

The distribution of percent body fat (PBF) was approximately normal, with *p* = 0.163 resulting from the Shapiro–Wilk test (Figure S1). In the insulin resistance group, the survival group had significantly higher percent body fat than the death group, with means of 31.3 ± 9.0 vs. 25.4 ± 8.2, *p* = 0.005, respectively (Table 1).

In the total sample, higher Charlson comorbidity index (hazard ratio, HR, 1.37; 95% confidence interval, 95% CI, 1.12–1.66; *p* = 0.002), cardiomegaly (or elevated CTR) (HR, 1.85; 95% CI, 1.02–3.34; *p* = 0.043), inflammation (or high hs-CRP) (HR, 2.98; 95% CI, 1.67–5.32; *p* < 0.001), and impaired fasting glucose (HR, 2.46; 95% CI, 1.15–5.29; *p* = 0.021) significantly increased all-cause mortality. Whereas, higher serum albumin (HR, 0.37; 95% CI, 0.18–0.74; *p* = 0.005), higher serum creatinine (HR, 0.81; 95% CI, 0.70–0.94; *p* = 0.005), and higher serum uric acid (HR, 0.75; 95% CI, 0.61–0.92; *p* = 0.005) significantly reduced all-cause mortality (Table 2).

Percent body fat showed a significant protective effect on survival in patients with insulin resistance via bivariate Cox proportional hazard model (HR, 0.94; 95% CI, 0.90–0.99; *p* = 0.017; Table 2), and multivariate Cox proportional hazard model (HR, 0.94; 95% CI, 0.89–1.00; *p* = 0.033; Table 3). In addition, an elevated level of intact parathyroid hormone was significantly associated with lower risk of death in patients with insulin resistance (HR, 0.19; 95% CI, 0.05–0.66; *p* = 0.009; Table 3).

## 4. Discussion

The most important finding of the current study is that a higher percent body fat was significantly associated with lower risk for all-cause mortality in the overall sample, and in patients with elevated HOMA-IR. In previous studies, body fat (as measured by percent body fat, or fat tissue, or body mass index) has been found to be an independent predictor for longer survival in hemodialysis patients [8,38,39,40,41,42]. In addition, a low baseline PBF and fat loss over time significantly increases mortality in maintenance hemodialysis patients, as found in a previous study [43]. In the current study, the percent body fat, but not body mass index, was significantly associated with all-cause mortality. This might reflect that percent body fat (as a direct measurement of body fat) is more accurate than body mass index (which cannot distinguish lean body mass and fat mass) in predicting all-cause mortality. One previous study in Japan has also reported that fat tissue index is more accurate than body mass index in determining all-cause mortality in hemodialysis patients [41].

In the present study, in the overall sample, higher levels of serum albumin, creatinine, and uric acid were significantly associated with reduced all-cause mortality. Higher Charlson comorbidity index, cardiomegaly (elevated CTR), inflammation (high hs-CRP), and impaired fasting glucose were significantly associated with increased all-cause mortality in the bivariate analysis. However, in the multivariate analysis, among above factors, elevated hs-CRP remained the significant association with mortality. Lower levels of serum albumin and elevated CRP were well-reported predictors of all-cause mortality in a previous study [44].

Elevated levels of intact parathyroid hormone (iPTH) were significantly associated with lower risk of all-cause mortality. This finding is consistent with previous studies’ results that decreased iPTH is associated with increased cardiovascular mortality [45] and all-cause mortality in hemodialysis patients [45,46].

In addition, serum uric acid was significantly inversely associated with all-cause mortality in the bivariate analysis and attenuated in the multivariate analysis in the current study. This is contradictory to the finding in the general population that a high level of serum uric acid is a risk for cardiac death [47]. In hemodialysis patients, higher serum uric acid has been reported as a protective factor against all-cause and cardiovascular mortality [48,49,50,51]. In addition, serum uric acid predicts mortality in hemodialysis patients with diabetes [52]. In our study, higher serum uric acid was associated with a lower risk of all-cause mortality in patients with insulin resistance. Serum uric acid could then play a mortality predictive role in hemodialysis patients.

The study has a certain number of limitations. Firstly, observational bias may have existed during the follow-up. However, patients were recruited from seven hospitals which might increase the external validity of findings. Secondly, the medications being used were not assessed, which may affect the associations of studied variables. In addition, estimates made on the basis of so few events are not stable, and thus not generalizable. Future studies with a comprehensive assessment are suggested to assure the current findings, and deeply examine the mechanism. The future research should be conducted in larger population with different races and ethnicities as obesity paradox varies across race-ethnic populations [53]. Regardless of the limitations, the current study demonstrates strength with its objective measures of body composition, and clinical and biochemical values.

## 5. Conclusions

The current study presents an association between percent body fat and all-cause mortality in adequate dialysis patients with and without insulin resistance. The results show that higher percent body fat is associated with lower all-cause mortality, especially in patients with insulin resistance. Assessments of body composition and insulin resistance are important for identifying appropriate therapies which can further reduce mortality in hemodialysis patients.

## Figures and Tables

**Table 1:** Patients’ characteristics, clinical, biochemical parameters at the baseline.

Variables	Total (*N* = 365)	Total (*N* = 365)			HOMA-IR < 5.18 (*N* = 183)			HOMA-IR ≥ 5.18 (*N* = 182)		
		Survival (*N* = 319)	Death (*N* = 46)	*p* ^1^	Survival (*N* = 157)	Death (*N* = 26)	*p* ^1^	Survival (*N* = 162)	Death (*N* = 20)	*p* ^1^
Age ≥ 65 years	129 (35.3)	108 (33.9)	21 (45.7)	0.118	53 (33.8)	11 (42.3)	0.397	55 (34.0)	10 (50.0)	0.158
Gender, male	205 (56.2)	175 (54.9)	30 (65.2)	0.186	88 (56.1)	14 (53.8)	0.834	87 (53.7)	16 (80.0)	0.025
Hemodialysis vintage, year	5.8 ± 5.0	5.9 ± 5.1	4.7 ± 4.1	0.137	6.7 ± 5.4	4.1 ± 2.1	0.018	5.1 ± 4.6	5.6 ± 5.5	0.717
CCI	4.7 ± 1.6	4.6 ± 1.5	5.5 ± 1.6	<0.001	4.4 ± 1.5	5.3 ± 1.8	0.008	4.7 ± 1.5	5.7 ± 1.3	0.004
PA, MET-min/week	4886.1 ± 1887.1	4999.4 ± 1895.2	4100.5 ± 1643.6	0.002	4865.4 ± 2033.4	3871.1 ± 1701.3	0.019	5129.2 ± 1747.5	4398.8 ± 1556.7	0.076
Clinical parameters										
SBP ≥ 130 mmHg	297 (81.4)	257 (80.6)	40 (87.0)	0.298	131 (83.4)	21 (80.8)	0.737	126 (77.8)	19 (95.0)	0.071
DBP ≥ 85 mmHg	90 (24.7)	80 (25.1)	10 (21.7)	0.623	40 (25.5)	5 (19.2)	0.493	40 (24.7)	5 (25.0)	0.976
BMI ≥ 24.0 kg/m^2^	146 (40.0)	127 (39.8)	19 (41.3)	0.847	48 (30.6)	9 (34.6)	0.680	79 (48.8)	10 (50.0)	0.917
CTR ≥ 50%	144 (39.5)	118 (37.8)	26 (57.8)	0.011	55 (35.9)	13 (52.0)	0.126	63 (39.6)	13 (65.0)	0.030
TMM, kg	41.3 ± 9.3	41.1 ± 9.3	42.7 ± 9.9	0.272	41.0 ± 8.6	40.9 ± 11.8	0.988	41.1 ± 9.9	44.9 ± 6.2	0.097
BFM, kg	17.8 ± 8.0	18.0 ± 8.1	16.5 ± 7.2	0.233	15.6 ± 7.2	16.3 ± 7.8	0.643	20.3 ± 8.3	16.7 ± 6.4	0.063
PBF, %	28.4 ± 9.7	28.7 ± 9.7	26.1 ± 9.3	0.092	26.0 ± 9.7	26.7 ± 10.3	0.745	31.3 ± 9.0	25.4 ± 8.2	0.005
Biochemical parameters										
hs-CRP > 0.5 mg/dL	105 (28.8)	81 (25.4)	24 (52.2)	<0.001	30 (19.1)	16 (61.5)	<0.001	51 (31.5)	8 (40.0)	0.443
Anemia (Hgb < 11 g/dL)	209 (57.3)	178 (55.8)	31 (67.4)	0.137	80 (51.0)	18 (69.2)	0.084	98 (60.5)	13 (65.0)	0.697
FPG (mg/dL)	131.5 ± 58.2	131.1 ± 58.8	134.3 ± 54.8	0.725	104.4 ± 35.8	121.0 ± 49.5	0.041	156.9 ± 64.9	151.7 ± 57.6	0.732
IFG ^2^	253 (69.3)	215 (67.4)	38 (82.6)	0.037	72 (45.9)	18 (69.2)	0.027	143 (88.3)	20 (100.0)	
Insulin, µU/mL	16.7 (8.8–31.8)	16.9 (8.8–32.2)	14.5 (7.3–28.7)	0.386	8.8 (5.9–12.7)	7.6 (6.0–14.0)	0.886	32.0 (24.2–49.3)	30.4 (19.9–38.9)	0.412
Insulin ≥ 12.0 µU/mL)	234 (64.1)	205 (64.3)	29 (63.0)	0.872	46 (29.3)	9 (34.6)	0.584	159 (98.1)	20 (100.0)	
HOMA-IR ≥ 5.18	182 (49.9)	160 (50.8)	20 (43.5)	0.354						
TG ≥ 150 mg/dL)	143 (39.2)	127 (39.8)	16 (34.8)	0.514	34 (21.7)	10 (38.5)	0.063	93 (57.4)	6 (36.0)	0.020
Low HDL-C (<40 mg/dL for men, <50 mg/dL for women)	203 (55.6)	180 (63.2)	23 (52.3)	0.167	78 (53.1)	14 (56.0)	0.785	102 (73.9)	9 (47.4)	0.017
LDL-C ≥ 100 mg/dL)	179 (49.0)	158 (49.5)	21 (45.7)	0.623	82 (52.2)	15 (57.7)	0.605	76 (46.9)	6 (30.0)	0.151
TC ≥ 200 mg/dL	62 (17.0)	57 (17.9)	5 (10.9)	0.237	30 (19.1)	3 (11.5)	0.352	27 (16.7)	2 (10.0)	0.442
Dyslipidemia ^3^	299 (81.9)	365 (83.1)	34 (73.9)	0.131	121 (77.1)	20 (76.9)	0.987	144 (88.9)	14 (70.0)	0.018
Serum Ca > 9.5 mg/dL	132 (36.2)	119 (37.3)	13 (28.3)	0.233	58 (36.9)	9 (34.6)	0.820	61 (37.7)	4 (20.0)	0.120
Serum PO_4_ > 5.5 mg/dL	126 (34.5)	113 (35.4)	13 (28.3)	0.339	57 (36.3)	8 (30.8)	0.585	56 (34.6)	5 (25.0)	0.392
Ca x PO_4_ ≥ 55 mg^2^/dL^2^	92 (25.2)	80 (25.1)	12 (26.1)	0.883	37 (23.6)	8 (30.8)	0.430	43 (26.5)	4 (20.0)	0.528
iPTH ≥ 300 pg/mL	157 (43.0)	142 (44.5)	15 (32.6)	0.127	71 (45.2)	11 (42.3)	0.782	71 (43.8)	4 (20.0)	0.041
Hcy > 14 µmol/L	314 (86.0)	276 (86.5)	38 (82.6)	0.474	133 (84.7)	21 (80.8)	0.610	143 (88.3)	17 (85.0)	0.672
Albumin, g/dL	4.0 ± 0.4	4.0 ± 0.4	3.9 ± 0.4	0.138	4.1 ± 0.3	3.9 ± 0.4	0.032	3.9 ± 0.4	3.9 ± 0.4	0.746
Pre-BUN, mg/dL	72.9 ± 19.4	72.7 ± 19.8	74.4 ± 16.0	0.574	71.9 ± 21.0	74.0 ± 16.7	0.626	73.4 ± 18.6	74.9 ± 15.4	0.737
Creatinine, mg/dL	11.0 ± 2.1	11.1 ± 2.1	10.4 ± 1.7	0.017	11.2 ± 1.9	10.1 ± 1.6	0.004	11.1 ± 2.4	10.7 ± 1.8	0.482
Hyperkalemia (K ≥ 5.0 mEq/L)	130 (35.6)	114 (35.7)	16 (34.8)	0.899	66 (42.0)	11 (42.3)	0.979	48 (29.6)	5 (25.0)	0.667
Uric acid, mg/dL	7.3 ± 1.2	7.3 ± 1.2	6.9 ± 1.2	0.021	7.2 ± 1.2	6.9 ± 1.3	0.316	7.4 ± 1.2	6.8 ± 0.9	0.025

CCI, Charlson comorbidity index; PA, physical activity; MET, metabolic equivalent minute/week; SBP, systolic blood pressure; DBP, diastolic blood pressure; BMI, body mass index; CTR, cardiothoracic ratio; TMM, total muscle mass; BFM, body fat mass; PBF, percent body fat; CRP, high-sensitivity C-reactive protein; Hgb, hemoglobin; FPG, fasting plasma glucose; IFG, impaired fasting glucose; HOMA-IR, homeostatic model assessment of insulin resistance; TG, triglyceride; HDL-C, high density lipoprotein cholesterol; LDL-C, low density lipoprotein cholesterol; TC, total cholesterol; Ca, serum calcium; PO_4_, serum phosphate; iPTH, intact parathyroid hormone; Hcy, homocysteine; Pre-BUN, pre-dialysis blood urea nitrogen; K, serum potassium. ^1^ Data was presented as mean ± SD, median (interquartile range), and percentage for normal distributed, non-normal distributed continuous variables, and categorical variables, respectively. *p* values calculated using independent-samples T test, Mann-Whitney U test, or Chi-square test, respectively. ^2^ Patients diagnosed as impaired fasting glucose when they had fasting plasma glucose ≥100 mg/dL or diagnosed with type 2 diabetes mellitus. ^3^ Patients were classified as dyslipidemia when they had high TG, or high LDL-C, or high TC, or low HDL-C.

**Table 2:** Hazard ratio of all-cause mortality among hemodialysis patients via simple logistic regression analysis.

Variables	Overall (*N* = 365)		HOMA-IR < 5.18 (*N* = 183)		HOMA-IR ≥ 5.18 (*N* = 182)	
	HR (95% CI)	*p*	HR (95% CI)	*p*	HR (95% CI)	*p*
Age ≥ 65 years	1.58 (0.88–2.82)	0.125	1.32 (0.61–2.88)	0.481	1.95 (0.81–4.69)	0.138
Gender, male	1.78 (0.97–3.28)	0.063	1.20 (0.55–2.63)	0.646	2.83 (0.94–8.50)	0.063
Hemodialysis vintage, year	0.95 (0.88–1.02)	0.142	0.88 (0.79–0.99)	0.028	1.01 (0.93–1.09)	0.896
CCI	1.37 (1.12–1.66)	0.002	1.32 (1.04–1.68)	0.023	1.40 (1.00–1.95)	0.049
PA, MET-min/week	0.95 (0.85–1.06)	0.368	0.93 (0.81–1.07)	0.313	0.99 (0.85–1.17)	0.942
Clinical parameters						
SBP ≥ 130 mmHg	1.78 (0.75–4.20)	0.190	0.95 (0.36–2.53)	0.923	6.71 (0.89–50.42)	0.064
DBP ≥ 85 mmHg	1.00 (0.49–2.01)	0.992	0.83 (0.31–2.20)	0.703	1.33 (0.48–3.69)	0.590
BMI ≥ 24.0 kg/m^2^	1.08 (0.60–1.94)	0.810	1.19 (0.53–2.66)	0.679	0.99 (0.41–2.38)	0.984
CTR ≥ 50%	1.85 (1.02–3.34)	0.043	1.47 (0.67–3.24)	0.338	2.66 (1.06–6.67)	0.037
TMM, kg	1.02 (0.99–1.05)	0.249	1.01 (0.97–1.05)	0.739	1.02 (0.98–1.06)	0.350
BFM, kg	0.98 (0.94–1.02)	0.227	1.01 (0.96–1.06)	0.796	0.94 (0.88–1.00)	0.062
PBF, %	0.97 (0.94–1.00)	0.088	1.00 (0.96–1.04)	0.982	0.94 (0.90–0.99)	0.017
Biochemical parameters						
hs-CRP > 0.5 mg/dL	2.98 (1.67–5.32)	<0.001	5.38 (2.44–11.85)	<0.001	1.39 (0.57–3.41)	0.468
Anemia (Hgb < 11 g/dL)	1.41 (0.76–2.60)	0.280	1.80 (0.78–4.15)	0.167	1.06 (0.42–2.66)	0.901
IFG ^1^	2.46 (1.15–5.29)	0.021	2.44 (1.06–5.60)	0.036	-	
Insulin ≥ 12.0 µU/mL)	1.14 (0.62–2.09)	0.669	1.27 (0.57–2.86)	0.561	-	
HOMA-IR ≥ 5.18	0.94 (0.52–1.69)	0.839	-		-	
Dyslipidemia ^2^	0.54 (0.28–1.04)	0.067	0.94 (0.38–2.34)	0.896	0.19 (0.07–0.51)	0.001
Serum Ca > 9.5 mg/dL	0.78 (0.41–1.49)	0.453	1.03 (0.46–2.32)	0.937	0.49 (0.16–1.47)	0.204
Serum PO_4_ > 5.5 mg/dL	0.73 (0.38–1.41)	0.351	0.79 (0.34–1.82)	0.584	0.51 (0.19–1.42)	0.200
Ca x PO_4_ ≥ 55 mg^2^/dL^2^	1.00 (0.52–1.94)	0.995	1.41 (0.62–3.26)	0.415	0.59 (0.20–1.78)	0.350
iPTH ≥ 300 pg/mL	0.62 (0.34–1.15)	0.129	0.96 (0.44–2.10)	0.927	0.29 (0.10–0.88)	0.029
Hcy > 14 µmol/L	0.76 (0.35–1.62)	0.473	0.80 (0.30–2.12)	0.650	0.64 (0.19–2.18)	0.471
Albumin, g/dL	0.37 (0.18–0.74)	0.005	0.29 (0.12–0.74)	0.009	0.43 (0.14–1.32)	0.138
Pre-BUN, mg/dL	0.99 (0.98–1.01)	0.420	1.00 (0.98–1.02)	0.708	0.99 (0.97–1.01)	0.348
Creatinine, mg/dL	0.81 (0.70–0.94)	0.005	0.76 (0.63–0.93)	0.006	0.83 (0.66–1.05)	0.114
Hyperkalemia (K ≥ 5.0 mEq/L)	0.81 (0.44–1.48)	0.488	0.89 (0.41–1.94)	0.767	0.75 (0.27–2.08)	0.581
Uric acid, mg/dL	0.75 (0.61–0.92)	0.005	0.83 (0.63–1.09)	0.182	0.63 (0.43–0.91)	0.015

HR, hazard ratio; CI, conference interval; CCI, Charlson comorbidity index; PA, physical activity; MET, metabolic equivalent minute/week; SBP, systolic blood pressure; DBP, diastolic blood pressure; BMI, body mass index; CTR, cardiothoracic ratio; TMM, total muscle mass; BFM, body fat mass; PBF, percent body fat; hs-CRP, high-sensitivity C-reactive protein; Hgb, hemoglobin; IFG, impaired fasting glucose; HOMA-IR, homeostatic model assessment of insulin resistance; Ca, serum calcium; PO_4_, serum phosphate; iPTH, intact parathyroid hormone; Hcy, homocysteine; Pre-BUN, pre-dialysis blood urea nitrogen; K, serum potassium. ^1^ Patients diagnosed as impaired fasting glucose when they had fasting plasma glucose ≥100 mg/dL or diagnosed with type 2 diabetes mellitus. ^2^ Patients were classified as dyslipidemia when they had high TG, or high LDL-C, or high TC, or low HDL-C.

**Table 3:** Effect of percent body fat on all-cause mortality in hemodialysis patients via multivariate logistic regression analysis.

	Overall (*N* = 365)		HOMA-IR < 5.18 (*N* = 183)		HOMA-IR ≥ 5.18 (*N* = 182)	
	HR (95% CI)	*p*	HR (95% CI)	*p*	HR (95% CI)	*p*
PBF, %	0.95 (0.92–0.99)	0.006	0.96 (0.92–1.01)	0.090	0.94 (0.89–1.00)	0.033
Hemodialysis vintage, year	-		0.92 (0.82–1.03)	0.154	-	
CCI	1.20 (0.98–1.48)	0.082	1.11 (0.86–1.44)	0.411	1.20 (0.86–1.67)	0.289
CTR ≥ 50%	1.71 (0.94–3.10)	0.080			1.89 (0.68–5.23)	0.222
hs-CRP > 0.5 mg/dL	3.08 (1.63–5.82)	0.001	4.64 (1.95–11.05)	0.001	-	
IFG ^1^	2.14 (0.96–4.80)	0.065	1.71 (0.68–4.29)	0.255	-	
Dyslipidemia ^2^	-		-		0.36 (0.11–1.19)	0.094
iPTH ≥ 300 pg/mL	-		-		0.19 (0.05–0.66)	0.009
Albumin, g/dL	0.89 (0.42–1.88)	0.765	1.00 (0.36–2.78)	0.998	-	
Creatinine, mg/dL	0.85 (0.72–1.02)	0.073	0.81 (0.64–1.01)	0.058	-	
Uric acid, mg/dL	0.82 (0.63–1.07)	0.138	-		0.78 (0.54–1.13)	0.186

HR, hazard ratio; CI, conference interval; HOMA-IR, homeostatic model assessment of insulin resistance; PBF, percent body fat; CCI, Charlson comorbidity index; CTR, cardiothoracic ratio; hs-CRP, high-sensitivity C-reactive protein; IFG, impaired fasting glucose; iPTH, intact parathyroid hormone. ^1^ Patients diagnosed as impaired fasting glucose when they had fasting plasma glucose ≥100 mg/dL or diagnosed with type 2 diabetes mellitus. ^2^ Patients were classified as dyslipidemia when they had high TG, or high LDL-C, or high TC, or low HDL-C.

## Supplementary Materials

The following are available online at https://www.mdpi.com/2072-6643/11/6/1304/s1, Figure S1: Distribution of percent body fat in hemodialysis patients.
