# Supplementary figures and images for: Impact of Percent Body Fat on All-Cause Mortality among Adequate Dialysis Patients with and without Insulin Resistance: A Multi-Center Prospective Cohort Study
Source: Nutrients. 2019 Jun 9;11(6):1304. doi: 10.3390/nu11061304 (PMC6627844; doi:10.3390/nu11061304)

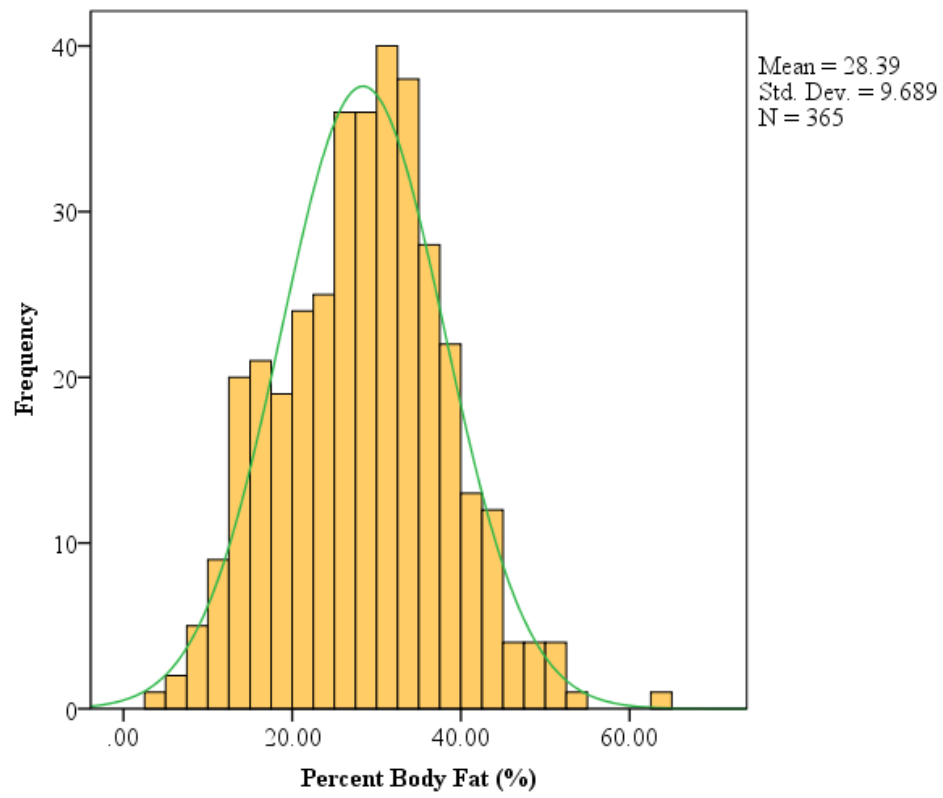

**Figure S1:** Distribution of percent body fat in hemodialysis patients

Supplement: Supplementary file 1 [file nutrients-11-01304-s001.pdf]
